# Clival Fracture With Multiple Cranial Nerve Palsy Treated With a Halo Device: A Case Report and Literature Review

**DOI:** 10.7759/cureus.69120

**Published:** 2024-09-10

**Authors:** Makoto Murase, Shinichi Yasuda, Tadashi Yahata, Koichi Inokuchi, Makoto Sawano

**Affiliations:** 1 Department of Emergency Medicine and Critical Care, Saitama Medical Center, Saitama Medical University, Kawagoe, JPN

**Keywords:** clivus, collet-sicard syndrome, external fixation, fracture, halo device, vernet syndrome

## Abstract

We present a rare case of clival fracture in which the patient presented with multiple lower cranial nerve palsy (similar to Vernet syndrome or Collet-Sicard syndrome). Multiple lower cranial nerve palsy from skull base lesions includes Vernet syndrome and Collet-Sicard syndrome. Clival fracture is a rare condition, and the optimal treatment method has yet to be established. A 73-year-old man fell down a flight of stairs and was diagnosed with injuries such as traumatic intracranial hemorrhage, clival fracture, and facial bone fracture. The patient presented with bilateral cranial nerve VI, IX, X, XI, and right XII palsies caused by clival fracture but no instability at the craniocervical junction, so we performed external fixation using a halo device. The patient developed delirium in the subacute phase, which was considered to be aggravated by the halo device. We therefore safely removed the halo device on hospital day 54 after confirming porosis of the fractured clivus on computed tomography. The patient did not complain of neck pain, and paralyses of cranial nerves VI and XII had completely resolved, while those of cranial nerves IX, X, and XI had also improved, so the patient could start direct swallowing training. Clival fracture with bilateral multiple cranial nerve palsies similar to Vernet syndrome or Collet-Sicard syndrome is highly rare. Accordingly, no standard treatment has been established. A halo device can be an effective treatment for clival fracture, and the duration of fixation could be determined flexibly based on the condition of each case.

## Introduction

Clival fracture is rare, accounting for 0.21%-0.56% of head injuries and 1.4%-2.3% of skull fractures [1-5]. Cranial nerve palsy is a common complication of clival fracture, seen in 13.8%-100% of cases [2-5], with cranial nerve VI and VII palsies being particularly common [3,6]. Lower nerve palsy due to clival fracture is notably rare, with no occurrences reported in some case series of clival fracture and few in case reports [6-12]. The optimal treatment for clival fracture has yet to be established, but internal or external fixation using a halo device has been described [9,10,13-19]. Several articles on external fixation with a halo device for clival and/or occipital condyle fracture have reported a fixation period of 12-18 weeks [9,10,16,20].

Vernet syndrome is defined as palsy involving cranial nerves IX, X, and XI, while Collet-Sicard syndrome is defined as unilateral palsy involving cranial nerves IX, X, XI, and XII [21,22]. These syndromes caused by a lesion adjacent to the skull base are relatively rare, and only one case appears to have been described in which Collet-Sicard syndrome occurred bilaterally [6].

Herein, we report a case of clival fracture with bilateral Vernet syndrome-like or Collet-Sicard syndrome-like multiple cranial nerve palsies that was successfully treated using external fixation with a halo device. In this context, we discuss treatments for clival fracture and review the literature.

## Case presentation

**Figure 1:**
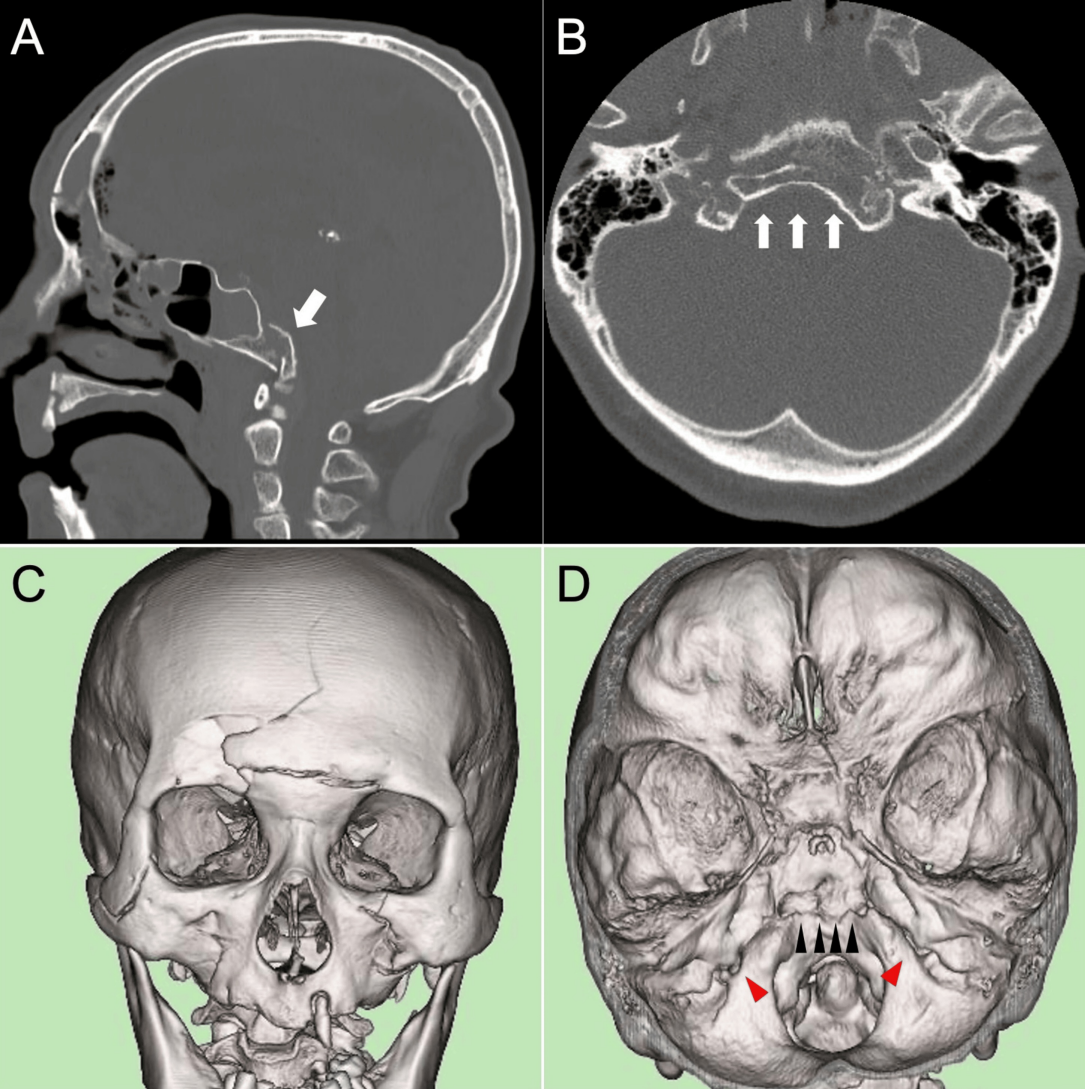
Imaging findings on the initial bone-window computed tomography (CT) in sagittal (A) and axial (B) views and three-dimensional (3D) reconstruction of CT of the face (C) and skull base (D). A: White arrow indicates the clival fracture and the entire head moves downward from its original position. B: The displaced fractured bone fragment is indicated by white arrows. C: The 3D reconstructed facial bone image from the front view shows multiple depressed facial bone fractures, suggesting the impact force was applied directly in front of the face. D: The fracture line is indicated with black arrowheads in the 3D skull base reconstruction from a posterior superior view. Red arrowheads indicate jugular foramina narrowed by the displaced fractured bone fragments.

A 73-year-old man fell down a flight of stairs and was brought to another hospital. The patient was diagnosed with traumatic subarachnoid hemorrhage, skull base fracture, frontal bone fracture, facial bone fracture, and left fifth finger fracture and was transferred to our institution for treatment. On physical examination, the patient was alert and showed no abnormalities in vital signs. However, the patient had hoarseness and difficulty in phonation and abduction was absent in both of the eyes. Computed tomography (CT) performed at our institution revealed traumatic subarachnoid hemorrhage, acute subdural hematoma, clival fracture, frontal bone fracture, pneumocephalus, right zygomatic fracture, ethmoid fracture, bilateral maxillary fractures, fractures of the 4th and 5th cervical spinous processes, and left fifth finger metacarpal fracture (Figure 1).

**Figure 2:**
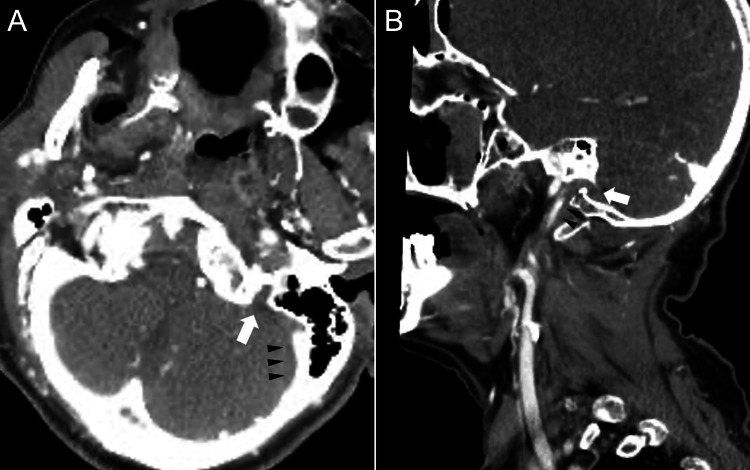
Computed tomography angiography on hospital day one (day of admission) in axial (A) and sagittal (B) views. White arrows indicate the left jugular foramen narrowed by the displaced fractured bone fragment in A and B. Black arrowheads in A indicate the sigmoid sinus with stagnant flow by the narrowed jugular foramen, while black arrowheads in B indicate the internal jugular vein.

Frontal fracture, ethmoid fracture, and bilateral maxillary fractures did not affect the extraocular muscles, suggesting that the patient’s inability to abduct was caused by bilateral abducens nerve palsy. Although the vertebral arteries appeared intact on CT angiography and venography performed at the same time, a fragment of fractured clivus was displaced to block both of the jugular foramina, resulting in occlusion of the left internal jugular vein (Figure 2).

**Figure 3:**
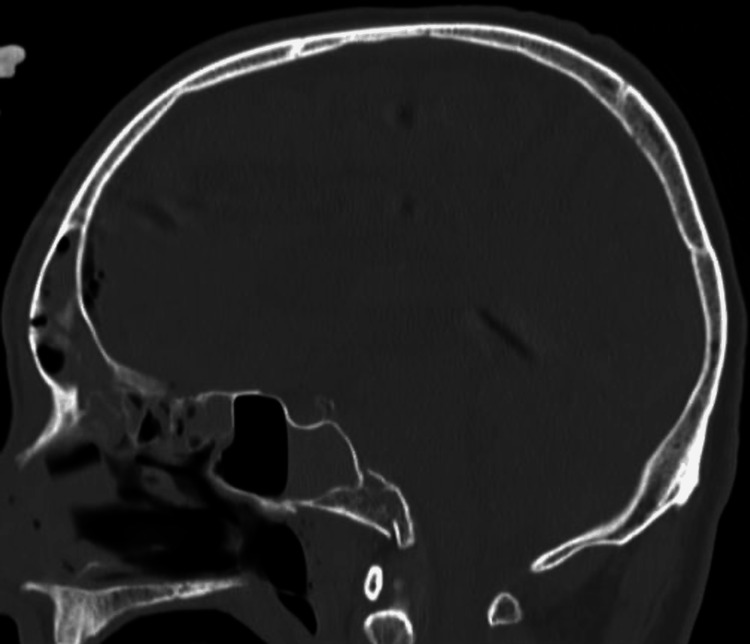
Sagittal-view bone-window computed tomography after reducing fracture of the clivus on hospital day two. Although the entire head was repositioned by about 2 mm, incarceration of the fractured bone fragment was not released.

Because of strong complaints of back neck pain from the clival fracture, we placed the patient in a halo ring to perform reduction and traction of the fracture. In the early hours of hospital day two, the patient required emergency intubation due to narrowing of the upper airway, which at the time, was thought to be caused by bleeding from the facial bone fracture. Although CT on hospital day two showed that the displaced bony fragment had returned about 2 mm upward, the incarceration had not been released (Figure 3).

**Figure 4:**
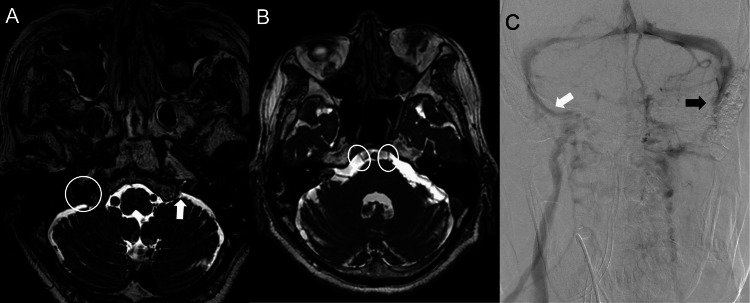
The heavily T2-weighted magnetic resonance imaging (A and B) and catheter-guided angiography (C) on hospital day two. A: White arrow indicates the left jugular foramen narrowed by the displaced fractured bone fragment, disrupting the lower cranial nerves running into the jugular foramen. White circle indicates the contralateral jugular foramen, which is slightly narrowed by the fractured bone fragment. B: White circles indicate the abducens nerves, which appear morphologically intact, and the structures around them are preserved. It implies that bilateral abducens nerve palsy was caused by stretching force at the time of injury. C: While the right internal jugular vein remains patent, the left sigmoid sinus (black arrow) is stagnant and the left jugular vein is almost unidentifiable. Blood flow in the sigmoid sinus drains from the emissary veins to the scalp veins.

The patient was then extubated after bleeding from the facial bone fracture stopped, but soon developed upper airway obstruction and was intubated again. Endoscopic examination at that time revealed bilateral vocal cord paralysis, which was considered to have contributed to the initial airway compromise. Magnetic resonance imaging (MRI) and catheter-guided angiography to again evaluate vertebral arteries and rule out brain infarction revealed no findings of intimal injury in either vertebral artery, or brain infarction, or any change in obstruction of the left internal jugular vein, but displaced fragments of fractured clivus compressed the lower nerves posteriorly (Figure 4).

**Figure 5:**
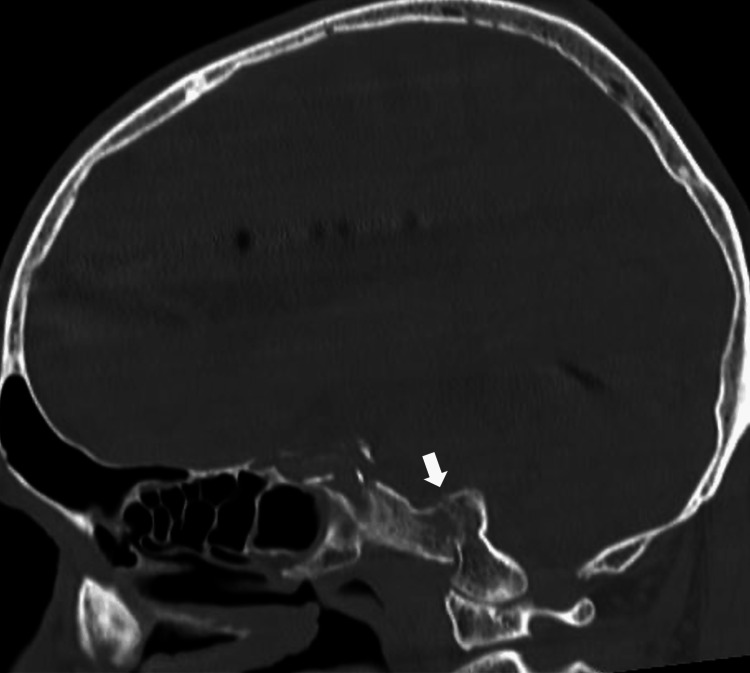
Sagittal-view bone-window computed tomography on hospital day 43. White arrow indicates porosis in the fractured clivus and consolidation of it, which suggests that the fracture is in the recovery process and becoming stable.

Palsy of bilateral cranial nerves IX, X, and XI were revealed on physical examination at that time, and we then placed the patient in a halo vest. On hospital day four, we performed a tracheostomy and reduced the clival fracture again. Right cranial nerve XII palsy was revealed on physical examination on hospital day seven. Although the patient underwent rehabilitation for neurological symptoms, recovery of swallowing function was poor, necessitating a gastrostomy on hospital day 28. The patient developed delirium a month after injury and needed medications, but symptoms of agitation proved difficult to control. Termination of halo fixation as soon as possible was considered desirable because of the possibility that this treatment was aggravating the delirium. After confirming porosis at the fractured clivus and consolidation of the fracture on CT (Figure 5), we exchanged the halo device for a rigid collar on hospital day 54.

This collar was subsequently removed on hospital day 63. MRI on hospital day 60 showed neither morphological changes in either of the vertebral arteries nor recanalization of the left internal jugular vein. The patient was transferred to another hospital for further rehabilitation. At the time of transfer, palsies of cranial nerves VI and XII had completely resolved, while those of cranial nerves IX, X, and XI had improved to the extent that direct swallowing training could be performed.

## Discussion

Vernet syndrome and allied syndromes are thought to be caused by a thrombus in the jugular bulb at the level of the jugular foramen, primary or metastatic tumors of the skull base, meningitis of the skull base, neurosyphilis, tumor at the cerebellopontine angle, skull base fractures, inflammatory diseases, retropharyngeal abscess, or vascular lesions such as aneurysm [21]. The most common cause of Vernet syndrome is reportedly a metastatic tumor of the skull base [23], while the most common cause of Collet-Sicard syndrome is reported to be a tumor [22]. Given that only one report of bilateral development of these syndromes could be found in the literature [6], the present case with bilateral traumatic multiple cranial nerve palsies resembling Vernet syndrome or Collet-Sicard syndrome is considered highly rare.

Many case reports have described clival fracture, but few large-scale case series are available, so the pathology of clival fracture remains unclear. We thus conducted a database search using the PubMed terms “clivus fracture” and “clival fracture” and reviewed the literature. We only included case reports and case series written in English and excluded articles for which only abstracts were available. Sixty-six articles were extracted, and then one article was excluded because it was a Letter to the Editor. As a result, we reviewed 211 cases with clival fracture from the remaining 65 articles [1-19,24-68] (Appendix). Clival fractures in all available radiographic images were morphologically classified as longitudinal, transverse, or oblique [2]. The 211 cases comprised 81 longitudinal cases (38.4%), 57 transverse cases (27.0%), and 71 oblique cases (33.6%), consistent with previous relatively large-scale case series that reported transverse fracture as the least frequent type of clival fracture [4,37].

We identified 48 cases (22.7%) with cranial nerve palsy; in these, 24 cases (50%) were transverse, 14 cases (29.2%) were longitudinal, and 10 cases (20.8%) were oblique. That was consistent with previous reports that transverse clival fracture is more likely to be associated with cranial nerve palsy [2,8,48]. Cranial nerve palsy most commonly affected cranial nerve VI, which was observed in 31 of the 211 cases (14.7%), followed by cranial nerve VII in 25 cases (11.8%). The most vulnerable cranial nerve in clival fractures is cranial nerve VI [2,8,48,66], which was damaged in the present case. Injury to this nerve is thought to be caused by stretching force derived from bilateral impacts causing the petrous apex to rotate backward and inward [50,61], or hyperextension of the neck at the time of injury [10]. In the present case, the latter was suspected as a cause because no petrous bone fracture was detected, and the clival fracture was anatomically distant from VI. Previous reports have suggested that the force sustained by the brain at the time of injury stretches and paralyzes cranial nerve VI [25,69]. Similarly, in the present case, cranial nerve VI could be stretched and damaged by ligaments such as Gruber’s ligament because a force of injury strong enough to fracture the clivus was applied to the brainstem.

In the literature, few cases have been described in which the lower cranial nerves were affected as in the present case, with one longitudinal, one oblique, and six transverse cases, in which any of cranial nerves IX-XII were affected. Among the six cases of transverse fracture, the fracture was located in the upper half of the clivus in two cases and in the lower half in four cases. Lower nerve palsy tended to be common in cases with fractures in the lower half of the clivus, with three of the four cases accompanied by occipital condyle fracture. Although occipital condyle fracture is likely to be accompanied by lower nerve palsy [70], which could be explained by anatomical proximity, no occipital condyle fracture was detected in the present case. Given that the present case showed facial bone fractures, the mechanism of injury was deduced to be facial injury during the fall, resulting in hyperextension of the neck, leading to the clival fracture, bilateral cranial nerve VI palsy, and displaced bone fragment disrupting cranial nerves IX-XI near the jugular foramina and concurrent fracture near the hypoglossal canal damaging the right cranial nerve XII.

The optimal treatment for clival fracture has not yet been fully established, and some articles have reported internal fixation, external fixation by halo device, and rest with a rigid collar [4,9,13-18,20,30,38,62]. Among 211 cases, all five cases in which internal fixation was performed showed transverse fractures, and of the six cases in which external fixation using a halo device was performed, five showed transverse fractures, and one had an oblique fracture. In all cases, whether transverse or oblique, the fracture was located in the lower half of the clivus, and in almost all cases (excluding one case with a transverse fracture), the fractures were accompanied by occipital condyle fractures. Many reports have argued that fractures of the lower part of the clivus and occipital condyle disrupt the tectorial membrane and the attached alar ligament, resulting in instability of the cervical vertebrae and the need for fixations [14,16,17,20].

Although no reports in the literature appear to have compared internal fixation with external fixation and comparisons are difficult because of the insufficient number of cases, some articles have reported that neurological symptoms including cranial nerve palsy improved as in the present study, whether internal or external [9,10,13]. In addition, previous articles have reported improvement of neurological symptoms with conservative treatments such as a rigid collar [3,7,8,11,12,60,61,63], for example, cranial nerve VI paralysis improved in 12 of 15 cases (80%), implying that the neurological prognosis did not correlate with treatment method. One case report described occiput-to-C1 fusion for bilateral occipital condyle fractures to preserve rotational motion at C1-C2 and to overcome the disadvantages of internal fixation that mobility of the cervical vertebrae would be lost [17]. Another case report suggested that external fixation with halo bracing correlated with a mortality rate as high as 26% in elderly patients, but could preserve rotational mobility at the craniocervical junction and thus may benefit younger patients with adherence as long as the ligaments remain intact [9,71].

Concerning the period of external fixation with a halo device for clival and/or occipital condyle fracture, several reports have argued a duration of 12-18 weeks [9,10,16,20], but one case report described fixation of occipital condyle fractures for eight weeks after injury due to a lack of adherence to the treatment [72]. In that case, the halo device was successfully removed, and the patient was treated with a rigid collar for six weeks. In the present case, the patient was treated with external fixation using a halo vest because the fracture was not accompanied by a fracture of the occipital condyle, and the traction test at the time of reduction did not indicate instability at the craniocervical junction. We could not avoid terminating fixation due to the delirium that developed in the subacute phase, after confirming porosis at the fractured clivus on CT. Evers et al. advocated an algorithm comprising 12 weeks of external fixation with a halo device followed by an assessment of the craniocervical junction instability to determine the method and duration [20]. However, the present case suggests that the duration of external fixation with a halo device could be shortened beyond the levels advocated in previous reports. Further clinical research is needed to confirm this possibility.

## Conclusions

We have presented a case in which external fixation with a halo device was performed for clival fracture accompanied by bilateral multiple cranial nerve palsies. Clival fracture accompanied by bilateral neurological symptoms similar to Vernet syndrome or Collet-Sicard syndrome is highly rare, and optimal treatment has therefore yet to be established. Further accumulation of cases is required to unveil the pathology and to establish the standard treatment for clival fracture.

## Appendices

**Table 1:** Selected studies CI: Complete improvement, PI: Partial improvement, N/A: Not available, NI: No improvement

Author	Year	Publication type	Number of cases	Intervention	Cranial nerve deficit		
					Cranial nerve number	Outcome	Time to recovery
Romero-López et al. [13]	2023	Case report	1	Internal fixation	VI	CI	6 months
Laing et al. [24]	2022	Case report	1				
Dimou et al. [25]	2021	Case report	1		VI, VII	VI: PI, VII: N/A	3 months
Lam et al. [15]	2021	Case report	1	Internal fixation			
Fromm et al. [7]	2021	Case series	2		VI, VII, XII	VI, XII: N/A, VII: PI	12 months
					VI, VII	VI: CI, VII: PI	7 months
Aljuboori et al. [14]	2020	Case report	1	Internal fixation			
Lagrand et al. [26]	2020	Case report	1				
Chan et al. [9]	2020	Case report	1	Halo device + Rigid collar	XII	CI	18 weeks
Metayer et al. [27]	2019	Case report	1		III, V, VI	III, V: NI, VI: CI	6 months
Kanamori et al. [28]	2018	Case report	1				
Gulen et al. [29]	2018	Case report	1				
Ucler et al. [30]	2018	Case report	1	Rigid collar	XII	CI	3 months
Wang et al. [31]	2017	Case report	1				
Tohge et al. [32]	2017	Case report	1				
Walker et al. [33]	2017	Case report	1				
Kliesch et al. [34]	2017	Case report	1				
Hirayama et al. [35]	2016	Case report	1				
Yamamoto er al. [36]	2016	Case report	1				
Winkler-Schwartz et al. [37]	2015	Original article	65		VI, VII	N/A	
					III	N/A	
					VI, VIII	N/A	
					VI	N/A	
					IV, V	N/A	
					VI	N/A	
					VI, VII	N/A	
					II, III	N/A	
					VII, VIII	N/A	
Akar et al. [38]	2015	Case report	1	Rigid collar			
Samraj et al. [39]	2015	Case report	1				
Buyukkaya et al. [40]	2014	Original article	2				
Chung et al. [41]	2014	Case report	1				
Evers et al. [20]	2013	Case report	1	Halo device			
Grossbach et al. [42]	2013	Case report	1		III	NI	3 months
García-García et al. [43]	2012	Case report	1				
Wang et al. [44]	2012	Case report	1				
Fang et al. [45]	2012	Case report	1				
Kim et al. [12]	2012	Case report	1		VI, XII	VI: PI, XII: NI	3 months
Sen-Gupta et al. [46]	2012	Case report	1				
Ochalski et al. [5]	2011	Original article	16	Rigid collar			
Ono et al. [47]	2011	Case report	1				
Khanna et al. [48]	2010	Case report	1				
Ochalski et al. [4]	2009	Original article	41	Rigid collar			
Cho et al. [49]	2008	Case report	1				
Katsuno et al. [50]	2007	Case report	1		VI	NI	4 weeks
Dashti et al. [16]	2007	Case report	1	Halo device + Rigid collar			
Gjertsen et al. [51]	2007	Case report	1				
Mathews et al. [19]	2007	Case report	1	Halo device			
Maughan et al. [17]	2005	Case report	1	Halo device + Internal fixation			
Paterakis et al. [11]	2005	Case report	1		VI, VII, IX	VI: PI, VII: CI, IX: CI	1 months
Kaakaji et al. [52]	2004	Case report	1				
Menkü et al. [3]	2004	Original article	9		VI	CI	3 months
					III, VI	Death	
					II, VI, VII	II: NI, VI: CI, VII: PI	1 year
					VI	CI	
					III, VI, VII	III, VI: CI, VII: PI	N/A
					VII	Death	
					III, VI, VII	Death	
					III, VII	Death	
					VII	PI	3 months
Bala et al. [53]	2004	Case report	1				
de Melo et al. [54]	2003	Case report	1				
Portier et al. [55]	2002	Case report	1				
Bonilha et al. [56]	2002	Case report	1				
Zhu et al. [57]	2002	Case report	1				
Sato et al. [58]	2001	Case report	1				
Ogungbo et al. [59]	2001	Case report	1				
Taguchi et al. [60]	2000	Case report	1		III, VI	III, VI: CI	6 months
Khan et al. [61]	2000	Case report	1		III, VI, VII	III, VI: PI, VII: NI	3 months
Imamura et al. [62]	2000	Case report	1				
Tanabe et al. [10]	1999	Case report	1	Halo device	VI, IX, X	VI, IX, X: CI	Unknown
Carter et al. [63]	1998	Case report	1		II, III, VI, VII, VIII	II: PI, III, VI, VII: CI, VIII: NI	5 months
Corradino et al. [2]	1990	Original article	17		III, IV	N/A	
					V, VI, VII	N/A	
					VI	N/A	
					III, IV, VI, VII	N/A	
					II, III, IV, VI	N/A	
Jones et al. [18]	1990	Case report	1	Rigid collar + Internal fixation			
Guha et al. [65]	1989	Case report	1				
Joslyn et al. [1]	1988	Original article	11				
Anthony et al. [66]	1987	Case report	1				
Kapila et al. [67]	1985	Case report	1		VI, VII, VIII	N/A	
Meguro et al. [6]	1985	Case report	1		V, IX, X, XI, XII	V: N/A, IX, X, XI, XII: PI	Unknown
Sanders et al. [8]	1973	Case series	3		V, VI, VII	V, VI: NI, VII: CI	Unknown
					VI, VII	VI, VII: CI	Unknown
					III, IV, V, VI, VII, IX	III, V, VII: CI, IV, VI, IX: NI	Unknown
Sights Jr et al. [68]	1968	Case report	1				
Loop et al. [69]	1964	Case report	1				

## References

[1] Joslyn JN, Mirvis SE, Markowitz B (1988). Complex fractures of the clivus: diagnosis with CT and clinical outcome in 11 patients. Radiology.

[2] Corradino G, Wolf A, Mirvis S, Joslyn J (1990). Fractures of the clivus: classification and clinical features. Neurosurgery.

[3] Menkü A, Koç RK, Tucer B, Durak AC, Akdemir H (2004). Clivus fractures: clinical presentations and courses. Neurosurg Rev.

[4] Ochalski PG, Spiro RM, Fabio A, Kassam A, Okonkwo D (2009). Fractures of the clivus: a contemporary series in the computed tomography era. Neurosurgery.

[5] Ochalski PG, Adamo MA, Adelson PD, Okonkwo DO, Pollack IF (2011). Fractures of the clivus and traumatic diastasis of the central skull base in the pediatric population. J Neurosurg Pediatr.

[6] Meguro K, Rowed DW (1985). Traumatic aneurysm of the posterior inferior cerebellar artery caused by fracture of the clivus. Neurosurgery.

[7] Fromm J, Meuwly E, Wendling-Keim D, Lehner M, Kammer B (2021). Clival fractures in children: a challenge in the trauma room setting!. Childs Nerv Syst.

[8] Sanders BB, VanderArk GD (1973). Transverse fracture of the clivus. J Neurosurg.

[9] Chan JL, Cohen JD, Rahman SU, Perry TG, Tuchman A (2020). Motion preserving management of unstable traumatic clivus fracture extending through bilateral occipital condyles. J Clin Neurosci.

[10] Tanabe M, Watanabe T, Matsumoto S, Okamoto H, Shirakashi K (1999). Avulsion fracture of the anterior half of the foramen magnum involving the bilateral occipital condyles and the inferior clivus--case report. Neurol Med Chir (Tokyo).

[11] Paterakis KN, Karantanas AH, Hadjigeorgiou GM, Anagnostopoulos V, Karavelis A (2005). Retroclival epidural hematoma secondary to a longitudinal clivus fracture. Clin Neurol Neurosurg.

[12] Kim SH, Kim SW (2012). Sixth and twelfth cranial nerve palsies following basal skull fracture involving clivus and occipital condyle. J Korean Neurosurg Soc.

[13] Romero-López C, Torri JA, Butrón-Díaz C, Rocha-Romero S, Martín-Schrader I, Arteaga-Romero F (2023). Displaced transverse clival fracture: infrequent but mortal. J Clin Neurosci.

[14] Aljuboori Z, Sharma M, Andaluz N (2020). Contemporaneous avulsion fractures of the inferior clivus and bilateral occipital condyles with injury of the tectorial membrane. Surg Neurol Int.

[15] Lam KS, Carriço G, Fernandes FM, Nanni F, de La Torre Escobar C (2021). A rare case of bilateral occipital condyle fractures associated with inferior clivus separation fracture resulting in craniocervical dislocation: a case report and modification of the Anderson and Montesano classification is proposed. Acta Neurochir (Wien).

[16] Dashti R, Ulu MO, Albayram S, Aydin S, Ulusoy L, Hanci M (2007). Concomitant fracture of bilateral occipital condyle and inferior clivus: what is the mechanism of injury?. Eur Spine J.

[17] Maughan PH, Horn EM, Theodore N, Feiz-Erfan I, Sonntag VK (2005). Avulsion fracture of the foramen magnum treated with occiput-to-c1 fusion: technical case report. Neurosurgery.

[18] Jones DN, Knox AM, Sage MR (1990). Traumatic avulsion fracture of the occipital condyles and clivus with associated unilateral atlantooccipital distraction. AJNR Am J Neuroradiol.

[19] Mathews MS, Owen CM, Hasso AN, Binder DK (2007). Traumatic retropharyngeal pseudomeningocele with atlanto-occipital dislocation in a neurologically intact patient. Neuroradiol J.

[20] Evers JJ, Vieth VV, Hartensuer RR, Raschke MM, Vordemvenne TT (2013). Management of an extended clivus fracture: a case report. BMC Res Notes.

[21] Svien HJ, Baker HL, Rivers MH (1963). Jugular foramen syndrome and allied syndromes. Neurology.

[22] Aguilera-Pena MP, Castiblanco MA, Osejo-Arcos V (2023). Collet-Sicard syndrome: a scoping review. Neurosurg Rev.

[23] Braut T, Maršić M, Ravlić I (2021). Posttraumatic Vernet syndrome without fracture: a case report and short literature review. Medicine (Baltimore).

[24] Laing BR, Hedayat HS (2022). Basilar artery incarceration secondary to a longitudinal clivus fracture: a rare and favorable outcome of an often devastating injury. Surg Neurol Int.

[25] Dimou S, Alukaidey L, Nair G (2021). A case report of bilateral abducens palsy in the setting of clival fracture - recovery related to pathophysiological basis of injury. Neuroophthalmology.

[26] Lagrand TJ, Bruijnes VA, Van der Stouwe AM, Deckers EA, Mazuri A, Jacobs B (2020). Locked-in syndrome after traumatic basilar artery entrapment within a clivus fracture: a case report and review of the literature. Neurotrauma Rep.

[27] Metayer T, Emery E, Cogez J, Barbier C, Gaberel T (2019). Entrapment of the basilar artery within a clivus fracture: case report and literature review. Neurochirurgie.

[28] Kanamori F, Yamanouchi T, Kano Y, Koketsu N (2018). Endovascular intervention in basilar artery entrapment within the longitudinal clivus fracture: a case report. Neurol Med Chir (Tokyo).

[29] Gulen B, Serinken M, Karcioglu O, Kucukdagli OT (2018). Longitudinal clival fracture in a child: case report and review of the literature. Pediatr Emerg Care.

[30] Ucler N, Yucetas SC (2018). Occipital condyle fracture extending to the inferior part of the clivus. Pediatr Neurosurg.

[31] Wang A, Wainwright J, Cooper J, Tenner MS, Tandon A (2017). Basilar artery herniation into the sphenoid sinus secondary to traumatic skull base fractures: case report and review of the literature. World Neurosurg.

[32] Tohge R, Takahashi M (2017). Cerebrospinal fluid rhinorrhea and subsequent bacterial meningitis due to an atypical clival fracture. Intern Med.

[33] Walker A, Dos Santos MP, Glikstein R, Michaud J (2017). Fatal entrapment of the basilar artery in a longitudinal fracture of the clivus due to head injury: a case report and review of the literature. Acad Forensic Pathol.

[34] Kliesch S, Bauknecht C, Bohner G, Liebig T, Siebert E (2017). Traumatic basilar artery entrapment with patency of pontine perforators and absence of significant brainstem infarction: report of an unusual case. J Neurointerv Surg.

[35] Hirayama A, Komatsu F, Hotta K, Imai M, Oda S, Shimoda M, Matsumae M (2016). Endoscopic endonasal repair of cerebrospinal fluid leakage caused by a rare traumatic clival fracture. Neurol Med Chir (Tokyo).

[36] Yamamoto S, Toyota S, Nakamura H, Mori K, Taki T, Yoshimine T (2016). Traumatic entrapment of the vertebral artery demonstrated by a 3D angiographic study. World Neurosurg.

[37] Winkler-Schwartz A, Correa JA, Marcoux J (2015). Clival fractures in a level I trauma center. J Neurosurg.

[38] Akar Ö, Yaldiz C, Özdemir N, Yaman O, Dalbayrak S (2015). Isolated transverse clivus fracture without neurodeficit: case report and review of literature. Pol J Radiol.

[39] Samraj RS, Stalets EL (2015). Pediatric longitudinal clivus fracture: survival with minimal morbidity. J Intensive Care Med.

[40] Buyukkaya R, Buyukkaya A, Ozturk B, Sarıtas A (2014). Complex fracture of the clivus after head trauma. Am J Emerg Med.

[41] Chung SW, Park KR, Jung YS, Park HS (2014). Fracture of the clivus as an unusual complication of LeFort I osteotomy: case report. Br J Oral Maxillofac Surg.

[42] Grossbach AJ, Abel TJ, Menezes AH, Howard MA (2013). Transverse clival fracture associated with bilateral petrous fractures extending through the occipital bone. J Neurosurg.

[43] García-García J, Villar-Garcia M, Abad L, Segura T (2012). Brainstem infarct due to traumatic basilar artery entrapment caused by longitudinal clival fracture. Arch Neurol.

[44] Wang E (2012). Clival fracture with basilar artery laceration. Curr Probl Diagn Radiol.

[45] Fang J, Kuang L, Chen J, Wang Y, Chen R, Xiong K, Zhang W (2012). Posttraumatic basilar artery herniation associated with dissecting aneurysm formation: follow-up over 20 months. Cardiovasc Intervent Radiol.

[46] Sen-Gupta I, Daiga DA, Alberts MJ (2012). Teaching neuroimages: locked-in syndrome resulting from traumatic basilar artery occlusion following clivus fracture. Neurology.

[47] Ono H (2017). Atypical clival fracture due to minor trauma and cerebrospinal fluid rhinorrhea. Intern Med.

[48] Khanna P, Bobinski M (2010). Computed tomography and magnetic resonance imaging of a basilar artery herniation into the sphenoid sinus. Skull Base.

[49] Cho J, Moon CT, Kang HS, Choe WJ, Chang SK, Koh YC, Roh HG (2008). Traumatic entrapment of the vertebrobasilar junction due to a longitudinal clival fracture: a case report. J Korean Med Sci.

[50] Katsuno M, Yokota H, Yamamoto Y, Teramoto A (2007). Bilateral traumatic abducens nerve palsy associated with skull base fracture--case report. Neurol Med Chir (Tokyo).

[51] Gjertsen O, Nakstad PH, Pedersen HK, Josefsen R (2007). Traumatic aneurysm of the superior cerebellar artery. Interv Neuroradiol.

[52] Kaakaji R, Russell EJ (2004). Basilar artery herniation into the sphenoid sinus resulting in pontine and cerebellar infarction: demonstration by three-dimensional time-of-flight MR angiography. AJNR Am J Neuroradiol.

[53] Bala A, Knuckey N, Wong G, Lee GY (2004). Longitudinal clivus fracture associated with trapped basilar artery: unusual survival with good neurological recovery. J Clin Neurosci.

[54] de Melo PM, Kadri PA, de Oliveira JG, Suriano IC, Cavalheiro S, Braga FM (2003). Cervical epidural haematoma with clivus fracture: case report. Arq Neuropsiquiatr.

[55] Portier F, Salvan D, Duruisseau O, Herman P, Tran Ba Huy P (2002). A rare clival and sellar fracture with pneumatocephalus. Auris Nasus Larynx.

[56] Bonilha L, Fernandes YB, Mattos JP, Borges WA, Borges G (2002). Bilateral internuclear ophthalmoplegia and clivus fracture following head injury: case report. Arq Neuropsiquiatr.

[57] Zhu BL, Quan L, Ishida K, Taniguchi M, Oritani S, Fujita MQ, Maeda H (2002). Longitudinal brainstem laceration associated with complex basilar skull fractures due to a fall: an autopsy case. Forensic Sci Int.

[58] Sato S, Iida H, Hirayama H, Endo M, Ohwada T, Fujii K (2001). Traumatic basilar artery occlusion caused by a fracture of the clivus--case report. Neurol Med Chir (Tokyo).

[59] Ogungbo B, Sengupta R (2001). Traumatic fracture of the clivus and vermian contusion in a child. Br J Neurosurg.

[60] Taguchi Y, Matsuzawa M, Morishima H, Ono H, Oshima K, Hayakawa M (2000). Incarceration of the basilar artery in a longitudinal fracture of the clivus: case report and literature review. J Trauma.

[61] Khan N, Zumstein B (2000). Transverse clivus fracture: case presentation and significance of clinico-anatomic correlations. Surg Neurol.

[62] Imamura T, Kojima T, Yashiki M, Namera A (2000). Traumatic avulsion fracture of the occipital condyles and clivus: a case report. Leg Med (Tokyo).

[63] Carter DA, Mehelas TJ, Savolaine ER, Dougherty LS (1998). Basal skull fracture with traumatic polycranial neuropathy and occluded left carotid artery: significance of fractures along the course of the carotid artery. J Trauma.

[64] Guha A, Fazl M, Cooper PW (1989). Isolated basilar artery occlusion associated with a clivus fracture. Can J Neurol Sci.

[65] Anthony DC, Atwater SK, Rozear MP, Burger PC (1987). Occlusion of the basilar artery within a fracture of the clivus. Case report. J Neurosurg.

[66] Kapila A, Chakeres DW (1985). Clivus fracture: CT demonstration. J Comput Assist Tomogr.

[67] Sights WP Jr (1968). Incarceration of the basilar artery in a fracture of the clivus. Case report. J Neurosurg.

[68] Loop JW, White LE Jr, Shaw CM (1964). Traumatic occlusion of the basilar artery within a clivus fracture. Radiology.

[69] Sam B, Ozveren MF, Akdemir I, Topsakal C, Cobanoglu B, Baydar CL, Ulukan O (2004). The mechanism of injury of the abducens nerve in severe head trauma: a postmortem study. Forensic Sci Int.

[70] Sharma BS, Mahajan RK, Bhatia S, Khosla VK (1994). Collet-Sicard syndrome after closed head injury. Clin Neurol Neurosurg.

[71] Hanigan WC, Powell FC, Elwood PW, Henderson JP (1993). Odontoid fractures in elderly patients. J Neurosurg.

[72] Persad AR, Liu E, Wu A, Fourney DR (2023). Bilateral occipital condyle fracture with an avulsion fracture of the foramen magnum: nonoperative care guided by a traction test. Illustrative case. J Neurosurg Case Lessons.

